# Cereal Germination under Low Oxygen: Molecular Processes

**DOI:** 10.3390/plants11030460

**Published:** 2022-02-08

**Authors:** Eva María Gómez-Álvarez, Chiara Pucciariello

**Affiliations:** PlantLab, Institute of Life Sciences, Scuola Superiore Sant’Anna, 56127 Pisa, Italy; eva.gomez@santannapisa.it

**Keywords:** anoxia, barley, germination, *Hordeum* spp., hypoxia, *Oryza* spp., rice, submergence

## Abstract

Cereal crops can differ greatly in tolerance to oxygen shortage under germination and seedling establishment. Rice is able to germinate and elongate the coleoptile under submergence and anoxia. This capacity has been attributed to the successful use of starchy reserves through a molecular pathway activated by sugar starvation and low oxygen. This pathway culminates with the expression of α-amylases to provide sugars that fuel the sink organs. On the contrary, barley and wheat are unable to germinate under anoxia. The sensitivity of barley and wheat is likely due to the incapacity to use starch during germination. This review highlights what is currently known about the molecular mechanisms associated with cereal germination and seedling establishment under oxygen shortage with a special focus on barley and rice. Insights into the molecular mechanisms that support rice germination under low oxygen and into those that are associated with barley sensitivity may be of help for genetic improvement programs.

## 1. Introduction

Rainfall intensity and frequency influence the exposure of crops to flooding when precipitation exceeds the soil’s capacity to drain water. In the Mediterranean area, this can be crucial for cereals when the annual rainfall is abundant during the sowing time [1]. In fact, sensitive crops suffer from the stress generated by the low O_2_ state that occurs under water. Oxygen is necessary for respiration in order to produce energy, and a long period of hypoxia inevitably generates an energy crisis with subsequent limited growth and productivity [2].

Stress from flooding also leads to a reduction in the availability of carbon dioxide and to a hampered diffusion of ethylene from plants into their environment [3]. In parallel, low light intensity due to the turbidity of floodwaters also affects photosynthesis during complete submergence, significantly reducing ATP synthesis. In fact, O_2_ deprivation shifts ATP production from respiration to fermentation, with a considerable reduction in energy yield [4].

Hypoxia and anoxia are not restricted to environmental stress, but they can also affect specialized tissues in plants. Variation in O_2_ concentration can occur in tissues with high cell density and limited gas diffusion [5]. Recently, hypoxic states have been identified in Arabidopsis meristems of lateral root primordia and shoot apical meristems [6,7].

Seeds can experience hypoxia during germination [8]. The reactivation of the metabolism begins with water imbibition and rehydration, which lead to the seedling growth. Once active, the initial respiratory activity consumes the O_2_ content in the seed. The O_2_ supply to the embryo through the seed coat can be restricted. In fact, the seed coat can act as a physical barrier for the exchange of gas [8,9,10]. In the context of seed germination under hypoxia, this barrier may be crucial in separating the capacity to germinate from the capacity to establish the seedling under hypoxia.

Hypoxia can be particularly harmful during the plant’s initial developmental phases of seed germination and seedling establishment [11]. In these phases, an efficient use of seed reserves mitigates energy starvation and allows some growth under anaerobic germination. Since germination and seedling establishment rely on the mobilization of reserves from the endosperm in order to fuel the embryo, the availability of carbon sources is crucial in these phases. In fact, at this stage, the photosynthetic system is still inactive.

Among cereals, rice can germinate under submergence and elongate the coleoptile to reach the water surface [12]. Several studies investigated the pathway involved in the rice unique capacity to degrade starch and mobilize soluble sugars from the endosperm to sink organs under low O_2_. This ability has not been observed in other cereals. On the contrary, barley is considered a low-O_2_-sensitive species, lacking the enzymatic set for starch breakdown in this condition [13]. Moreover, hypoxia has been shown to promote secondary dormancy in barely, identifying the presence of a hormonal bottleneck to successful germination.

In this review, we give an overview of what is currently known about the capacity of cereals to germinate and establish seedlings under O_2_ shortage, with a special emphasis on barley and rice. We summarise the molecular mechanisms that contribute to sugar mobilization and hormonal regulation, providing comparisons aimed at a better understanding of the complex signaling network.

## 2. Rice Germinates under Anoxia and Submergence

Unlike other cereals, rice is able to germinate well under hypoxia and anoxia. In fact, rice harbours α-amylase genes, which respond to a pathway activated by low O_2_ and sugar starvation [14,15,16,17]. However, complete submergence due to extreme precipitation during germination and seedling establishment can be critical for the success of direct seeding in rice fields [18]. Water seeding reduces the labour costs of transplanting and the costs of weed control. Since many Asian rice varieties are poorly tolerant to flooding when at the early seedling stage [19], tolerant genotypes that exhibit rapid and uniform germination and rapid and robust coleoptile elongation can be extremely effective.

Germination is regulated by several hormones, with ABA and GA being the key antagonistic regulators [11]. In cereals, GA and sugar demand mediates the mobilisation of reserves in the endosperm [20]. The subsequent activation of α-amylases, the most abundant hydrolases, supports cleaving of starch toward the production of sugars to subsequently fuel sink organs [18].

In rice germination under aerobic conditions, following water imbibition, sugars are promptly used. Sugar demand activates the expression of α-amylases through the sugar response element (SRE) located on the promoter regulatory region of the gene, which is the target of the sugar starvation responsive R1 MYB (MYBS1) transcription factor [21,22]. Alpha-amylases are also activated by GA via the presence of a GA-response element (GARE) on the gene promoter, which is the target of the GA-inducible R2R3 MYB transcription factor MYBGA [23,24]. Alpha amylases degrade starch stored in the endosperm to soluble sugars that are moved to the embryo to sustain the growth of the seedling [25].

### 2.1. Rice Molecular Mechanism Finalized to Starch Degradation under Low Oxygen

Rice α-amylase genes are classified in three subfamilies, where subfamily 1 and 2 respond to GA, while sugar starvation and low O_2_ regulate subfamily 3 [11]. In rice germination under low O_2_, subfamily 3 is predominantly induced. The hydrolysis of starch occurs when the signals involved in starvation and low O_2_ state converge in the activation of GA-independent α-amylases [26]. GA is probably not produced under anoxia due to the requirement of O_2_ for the synthesis and the production of GA-active molecules [27,28]. 

**Figure 1:**
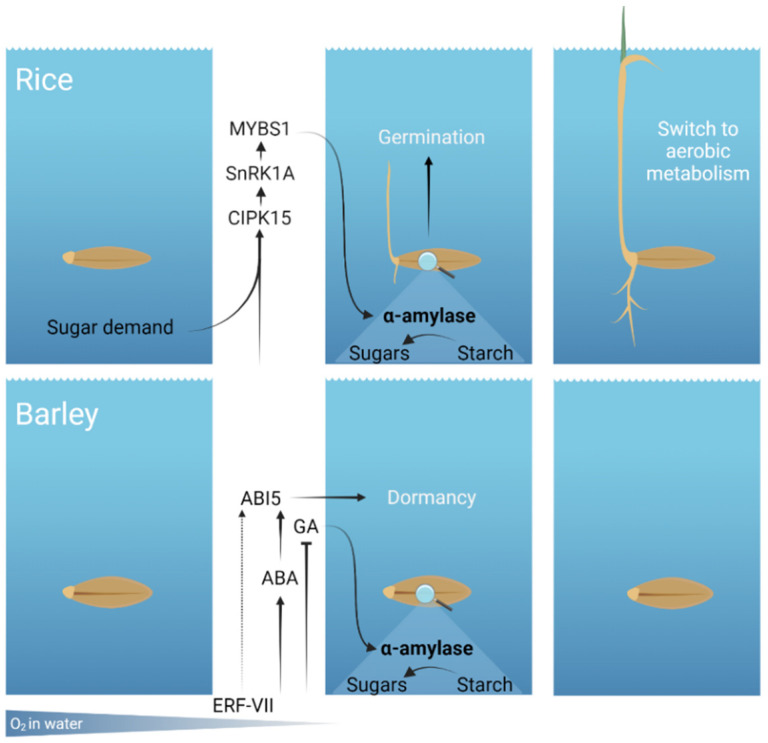
Possible mechanisms of rice and barley response to prolonged submergence. In rice, the anaerobic germination is regulated by hypoxia-dependent signaling and sugar starvation. The main upstream regulator of this pathway is CIPK15 which activates a signaling cascade culminating with expression of subfamily 3 α-amylase. In barley, GA biosynthesis is dampened under low O_2_ and ABA synthesis and signaling are promoted. As a consequence, GA-dependent α-amylases may not be expressed. Results obtained with Arabidopsis suggest that ERF-VIIs promote seed dormancy and ABA sensitivity through *ABI5* regulation. Image created with BioRender.com (accessed on 14 December 2021).

In rice, anaerobic germination is regulated by a pathway activated by sugar starvation and hypoxia-dependent Ca^2+^ signals (Figure 1). The main upstream positive regulator of this pathway is the calcineurin B-like protein (CBL)-interacting protein kinase (CIPK) CIPK15 that, together with a CBL Ca^2+^ sensor, contributes to the decoding of Ca^2+^ signal [29]. CIPK15 belongs to a group of plant-specific Ser/Thr protein kinases that harbour an N-terminal kinase catalytic domain and a self-inhibitory NAF/FISL motif. The NAF/FISL motif allows the interaction with CBL Ca^2+^ sensors [30]. Upon Ca^2+^ availability in the cytosol, CBLs undergo modifications that enable them to bind to CIPKs with the subsequent activation of the kinase. Experiments conducted on rice protoplasts have proposed CBL4 as a positive regulator of the CIPK15-dependent pathway, through the interaction with CIPK15 and the subsequent modification the downstream *α-Amy3* expression [31]. In parallel, a study of different rice genotypes identified CBL10 as a negative regulator of the CIPK15-dependent pathway [32]. In fact, the analysis of tolerant and sensitive rice cultivars *CBL10* promoters identified a correlation between promoter variations and flooding tolerance. The tolerant type promoter was likely responsible for a reduced expression of *CBL10* during germination under flooding and a subsequent higher *α-Amy3* expression and α-amylase activity. Moreover, rice *CBL10* overexpression lines were more sensitive to germination under flooding than wild type plants [32].

CIPK15 downstream events include the regulation of the sucrose-non-fermenting-1-related protein kinase 1A (SnRK1A) and the transcriptional activator MYBS1 [21,22,29]. MYBS1 binds to the promoter of subfamily 3 α-amylase, which is then expressed and is implicated in the starch hydrolysis in the rice endosperm. The R1 MYB transcription factor MYBS2 has also been found to play a role in regulating gene expression in response to the sugar status [33]. When sugar is available, MYBS2 functions as a repressor of *α-amylase* expression, competing for promoter binding with MYBS1. The *MYBS2* overexpression line showed reduced tolerance when seed germination occurred under submergence conditions. When wild-type plants were germinated under submergence, the expression of *MYBS2* was reduced in comparison to air. *MYBS2* expression did not vary in the *cipk15* mutant, suggesting that CIPK15 may downregulate MYBS2 under submergence.

In order to identify genotypes able to germinate under flooding, a phenotype screening was performed on a large panel of rice accessions [34]. Several QTLs were identified, including *qAG-9-2* available on chromosome 9 that contains the trehalose 6 phosphate phosphatase 7 (*TPP7*) gene, responsible for enhanced anaerobic germination tolerance [35]. A further regulation of source to sink sugar mobilisation during anaerobic germination is played by trehalose 6 phosphate (T6P), whose level, depending on local sucrose availability, plays a key role in the sugar flux to sink organs [36]. In this pathway, the availability in some rice genotypes of *TPP7*, which codifies for the enzyme that converts T6P in trehalose, modifies the T6P/sucrose balance. This is likely to result in an increase in the source to sink flux through the starch mobilization by α-amylases, which thus benefits seedling establishment under submergence [11,36].

The role of the phytoglobin/nitric oxide (Pgb/NO) cycle has been examined in relation to the ability of deepwater rice to germinate anaerobically [37]. The Pgb/NO cycle has been proposed to produce a small amount of ATP during O_2_ shortage [38]. This cycle includes the reduction of nitrate to nitrite by nitrate reductase in the cytosol. Subsequently, nitrite is translocated into the mitochondria and reduced to NO, allowing ATP generation. Finally, NO moves from the mitochondrial matrix back to the cytosol where it is oxidised to nitrate by Pgb. Interestingly, the supply of nitrite to the submergence water enhanced the capacity of deepwater rice to germinate under anoxia. Nitrite was shown to increase the production of both NO and ATP levels under anoxia, suggesting that the Pgb/NO cycle may contribute to energy availability in these conditions [37].

### 2.2. Rice Coleoptile Elongation under Low Oxygen

The translocation of sugars from source to sink initially aids coleoptile elongation (the conical structure that covers the emerging shoot), which in some *japonica* accessions is exceptionally long [39]. A long coleoptile enables the underwater organs to restore contact with the air and to initiate aerobic respiration. Interestingly, rice *japonica* accessions consume all the O_2_ available in water during coleoptile elongation [39]. Subsequently, when the coleoptile is in contact with the air, the full availability of energy leads to the development of the first leaf and the roots which is initially dampened [40].

*TPP7* gene has been shown to substantially contribute to the elongation of coleoptile since the near isogenic line NIL-AG1 (containing *qAG-9-2*) showed a significant increase in coleoptile length in comparison to the background [35]. In some rice *japonica* accessions, extreme coleoptile elongation is also regulated by the higher capacity to translocate auxins via AUX1, likely to favour the extensions of cells until the plateau length has been reached [41]. In this context, the elongation of rice coleoptiles under submergence is regulated by auxin-dependent signalling. In fact, the availability of the auxin receptors, transport inhibitor response 1 (TIR1) and auxin signalling F-box 2 (AFB2), is enhanced under submergence due to the repression of the microRNA *miR393* [42]. In Arabidopsis, microRNA miR393 degrades *TIR1* and *AFB2*, which are regulators of auxin responsive gene expression [43].

## 3. Barley Is Unable to Germinate under Anoxia and Prolonged Submergence

Barley is considered one of the most susceptible cereals to anaerobic stress [15,44]. An analysis comparing the germination capacity of several varieties of barley, durum and bread wheat under prolonged submergence revealed that barley is the most sensitive [45]. Under anoxia, barley is unable to germinate, likely due to the lack of α-amylases whose activation in rice is independent of GA [26] (Figure 1). In fact, no equivalents of subfamily 3 α-amylase have been found to be expressed under anoxia in barley and wheat [15]. Interestingly, rice contains four α-amylases belonging to family 3, while barley and wheat have only one each [46].

### 3.1. Hypoxia Affects Hormonal Regulation in Barley Grains

While barley is unable to germinate under anoxia, a large variability in germination capacity has been observed among varieties after short submergence periods [45]. When barley grains are exposed to a few days of hypoxia, they can experience secondary dormancy [47]. 

The seeds can be subjected to two types of dormancy. Primary dormancy is induced during seed development and is associated to germination inhibition when adequate environmental conditions are available [8,48]. Secondary dormancy is induced in mature seeds by adverse environmental conditions due to unfavourable temperatures, humidity, light, and O_2_ availability [49]. Oxygen limitation to the barley embryo is a regulator of germination, enhancing ABA sensitivity and GA inactivation [47,50].

Dormancy of the barley grain has been attributed to the structures that cover the seed, such as the seed coat, pericarp, lemma, and palea. In fact, dormant barley embryos can germinate well when isolated from the grain [51]. In addition, unlike caryopses, excised embryos can germinate under hypoxia, suggesting that covering structures may also reduce O_2_ availability for the embryo [52]. The limited O_2_ supply caused by the presence of the glumellae is not only due to the physical barrier but has also been suggested to be the result of highly active polyphenol oxidase that consumes O_2_ [53,54]. The limitation of O_2_ availability due to covering structures no longer has an effect after the radicle has protruded [8].

The removal of glumellae in barley seeds reduces the ABA increase, which happens after seed imbibition [55]. Glumellae and hypoxia both promote dormancy maintenance after imbibition. However, the mechanisms imposed by the hull and hypoxia seem to be different. At 30 °C, hull-imposed dormancy relies on a higher capacity to synthesise ABA through an increase in the expression of genes involved in ABA metabolism and signalling, such as *NCED1* and *ABI5*. This effect was not mimicked by hypoxia treatment of dehulled caryopses [55].

In barley embryos isolated from dormant grains, hypoxia at 15 °C induces the early expression of the *GA2ox3* gene, which is responsible for GA inactivation [47]. In parallel, there is a strong initial repression of the *GA3ox2* gene, which is responsible for GA synthesis. The upregulation of *NCED2*, involved in ABA biosynthesis, has also been observed [47].

### 3.2. Molecular Mechanisms Regulating Barley Sensitivity to Low Oxygen

The Pgb/NO cycle plays a further regulation role in barley’s response to brief episodes of submergence stress during germination. *Pgb1* is induced during barley germination, likely in line with the phase of hypoxia experienced by seeds after imbibition and due to the rapid use of O_2_ [56]. Under hypoxia, the activation of the NO turnover in the Pgb/NO cycle is an alternative to fermentation for the production of a limited quantity of ATP [38]. During germination, the production of NO in barley seeds starts immediately after the onset of imbibition [57].

NO is a powerful agent in breaking seed dormancy [58]. The application of the NO donor sodium nitroprusside (SNP) to dormant barley seeds has been shown to induce germination. On the other hand, 2-(4-carboxyphenyl)-4,4,5,5-tetramidazoline-1-oxyl-3 oxide (cPTIO), a NO scavenger, strengthens the dormancy in dormant barley seeds. Hypoxia induces Pgb, which scavenges NO to nitrate, which may restrict NO availability and exacerbate the dormancy during germination.

During germination, the overexpression of *Pgb1* in barley has been shown to increase the ATP/ADP ratio [56]. In parallel, the knock-down of *Pgb1* resulted in a strong increase in NO availability. Only barley grains overexpressing *Pgb1* were able to germinate under hypoxia [59], suggesting the importance of Pgb/NO cycle activation in this context.

Another aspect that may influence the capacity of barley to germinate under low O_2_ is the positive role played by reactive oxygen species (ROS), which are produced via NADPH oxidases after seed imbibition [60,61]. NADPH oxidases reduce O_2_ to superoxide, which is subsequently dismutated to hydrogen peroxide. Diphenylene iodonium chloride (DPI) is a potent inhibitor of NADPH oxidase activity, and it has been shown to dampen barley germination in a dose–response way. In fact, DPI application reduces the GA content in embryos but increases the ABA content. DPI also dampens the GA-dependent α-amylase activity in embryoless half-seeds during the first hours after the imbibition. In this context, hypoxia may reduce the substrate availability for NADPH oxidases, thus negatively regulating GA-dependent α-amylases.

Finally, several works have reported the accumulation of Ala under hypoxia [62,63,64]. The expression and enzyme activity of Ala aminotransferase (AlaAT) are also strongly up-regulated by hypoxia [65,66]. Under O_2_ shortage, the function of AlaAT is probably to maintain the glycolytic flux with the parallel storage of carbon and nitrogen resources within the cell [65]. AlaAT plays a central role in barley seed dormancy, with alleles differing in a single amino acid residue involved in long or short dormancy [67]. Hypoxia may thus have different impacts on the dormancy of barley seeds due to the different AlaAT isozymes available in the genotype.

## 4. Wheat Response to Anoxia and Submergence during Seed Germination

Wheat is not able to germinate under anoxia, probably due to its inability to express α-amylases and thus to break down starch [15,68]. Early results reported a rapid sugar starvation of the embryo but also the possibility of germination when wheat seeds were fed with exogenous glucose or sucrose [69]. The presence of starch in the endosperm thus does not itself ensure sugar availability for germination if it is not readily usable. Despite the massive starch reserves of wheat, it is unable to express α-amylase in response to O_2_ deprivation, suggesting the absence of a GA-independent α-amylase such as in barley.

An analysis of the capacity to convert carbohydrates to ethanol and CO_2_ under anoxia revealed that wheat and barley produce a similar amount of ethanol per seed to rice during the first days of anoxia, but subsequently this capacity is reduced [70]. Moreover, under anoxia wheat and barley use sucrose less efficiently than rice, supporting the greater capacity of rice to activate the anaerobic pathway. An analysis of the capacity of germination under submergence using different durum and bread wheat varieties suggested that after three days of stress, the percentage of germination is reduced considerably [45]. However, a few wheat varieties showed some levels of germination which were maintained for up to 15 days of treatment.

Spring wheat was analysed in terms of its capacity to germinate and its protein expression profile under submergence, which were compared with drought and salinity for up to three days after seeding [71,72]. Submergence was shown to be the most severe stress on germination. Wheat was not able to germinate under submergence and the analysis of protein accumulation showed a dampening of α-amylase together with enzymes involved in sucrose metabolism. A transcriptomic analysis of various wheat varieties germinated under water for three days showed differences in the expression of genes involved in glycolysis, starch, and sucrose metabolism between sensitive and tolerant varieties [73].

## 5. The N-Degron Pathway for Low Oxygen Sensing during Germination

The N-degron pathway controls the stabilisation of the ethylene response factors (ERFs) of group VII in plants in response to O_2_ availability [74]. Group VII ERFs are characterized by Met-Cys residues at the N-terminus, which render these proteins a substrate for degradation via the proteasome. In fact, Met is cleaved by Met aminopeptidases, revealing the Cys residue that is enzymatically oxidised by plant cysteine oxidases (PCOs) [75,76]. Subsequently, group VII ERFs are arginylated by argynil-transferases ATEs, and thereafter recognised by the E3 ligase PRT6 for degradation [77,78]. ERF-VIIs are also destabilised by NO via the N-degron pathway [79] through a mechanism that has not yet been fully elucidated. Interestingly, Arabidopsis PRT6 possesses a heme NO/O_2_ (H-NOX) domain that can operate as NO-binding, which suggests that it may play a role in the subsequent group VII ERFs regulation [80].

During Arabidopsis germination, the N-degron pathway promotes seed-to-seedling transition [81]. In fact, Arabidopsis mutants for *prt6* and the double mutants *ate1-2 ate2-1* show an extreme sensitivity to ABA, with a strong reduction in germination in the presence of an exogenous ABA treatment. This suggests that PRT6 and ATEs play a role in regulating ABA sensitivity during germination. In addition, analysis of the genetic relationship between PRT6 and components of the ABA pathway using single and double *prt6* and *abi* mutants combination suggested an interaction between PRT6 and ABA signalling, where the effect of *prt6* during germination is by-passed when ABA sensitivity is removed.

Together with a hypersensitivity to ABA, N-degron pathway mutants are not sensitive to the dormancy-breaking activity of NO [79]. Dormant Arabidopsis seeds were shown to germinate when treated with NO donors S-nitroso-*N*-acetyl-dl-penicillamide (SNAP) or SNP, while *prt6* and *ate1-2* were not. ERF-VIIs were shown to mediate the cross-talk between ABA and NO during germination. The quadruple mutant *prt6rap2.12rap2.2rap2.3* showed a reduction in dormancy and a lower sensitivity to ABA compared to the single mutant *prt6*. In line with these results, the expression of *RAP2.2, RAP2.12* and *RAP2.3* in *prt6* protoplasts was shown to induce GUS activity by a minimal *ABI5* promoter that contains two consensus binding sites for ERF-VIIs. In addition, chromatin immunoprecipitation showed that RAP2.3 physically interacts with the *ABI5* promoter region which contains the two ERF-VIIs binding sites [79].

The increase in NO and the availability of O_2_ during germination may thus promote ERFVIIs degradation with the subsequent downregulation of *ABI5*. In this sense, the presence of hypoxia stabilises ERF-VIIs with the subsequent regulation of *ABI5* possibly promoting dormancy. Chilling treatment under low O_2_ showed a better germination of *prt6* and *ate1-ate2* Arabidopsis mutants, on the contrary of unchilled seeds [55].

An analysis of the role of the N-degron pathway in barley under germination revealed that the reduced expression of *HvPRT6* obtained through RNAi results in seed germination impairment [82]. In addition, *Hvprt6* RNAi lines were more sensitive to the treatment with the NO scavenger cPTIO. These results indicate that the N-degron pathway substrate stabilisation, following hypoxia and NO scarcity, dampens the germination capacity of barley. Considering the results obtained with Arabidopsis, this may be related to ABA sensitivity.

## 6. Conclusions

Cereal crops differ in their capacity to successfully germinate under O_2_ shortage, and several works have examined the molecular basis that determines the capacity of rice and barley to face the hypoxia/anoxia stress during the germination stage (Table 1). A few data are also available for other cereals such as wheat. The fact that seeds may experience hypoxia as part of germination is challenging when this state occurs in a natural environment (e.g., submergence).

The phytohormones ABA and GA control germination antagonistically and represent the hub to decipher external stimuli for germination or dormancy. The investigations conducted on barley seeds strongly support the idea that hypoxia modifies the hormonal ABA-GA pattern activated under germination, thus modifying the state of dormancy. They also highlight that seed-covering structures exacerbate this phenomenon. These aspects have been mainly explored in barley germination under hypoxia, but very few studies have investigated this in rice. Application of GA and ABA in air showed a similar effect on the germination of the NIL-AGI1, harbouring the *TPP7* gene, and its background IR64 [35]. Moreover, continuous application of GA during anaerobic germination promotes coleoptile elongation of IR64 and NIL-AGI1 in a similar way, suggesting that TPP7 does not work through this hormonal regulation.

A crucial factor for cereal germination under O_2_ shortage is the capacity to use starchy reserves. The pathway that allows rice to use starch for the production of soluble sugar for germination has been widely studied. However, little is known about the direct molecular mechanisms that prevent barley from activating α-amylases in the seed endosperm under anoxia and the characteristics of α-amylases 3 in this species. This last aspect may be crucial to explain the absence of germination under anoxia.

A very intriguing aspect is the role of the N-degron pathway in the regulation of seed dormancy. Arabidopsis ABI5 has been proposed to be regulated by RAP type ERF-VIIs, thus contributing to hypoxia’s role in germination/dormancy. In barley, the N-degron pathway may also be involved in ABA sensitivity.

Rice ERF66 and ERF67, which belong to the ERF-VII group, are targets of the N-degron pathway [83]. Interestingly, these TFs have also been found to be transcriptionally regulated by SUB1A, which is not a target of the N-degron pathway. Currently, it is not known whether ERF66 and ERF67 influence the ABA-GA hormonal pattern; however, this aspect would be interesting to investigate given the excellent capacity of rice to germinate under O_2_ shortage.

The Pgb/NO cycle is also fascinating given that hypoxia induces Pgb, which scavenges NO, a powerful agent of dormancy breaking, thus possibly influencing dormancy and germination. However, the Pgb/NO cycle can help maintain a high energy state under hypoxia. The recent results obtained by manipulating *HvPgb1*, i.e., the capacity of barley to germinate under hypoxia when *Pgb1* is overexpressed [59], suggest that the source of energy is majorly important in this framework.

In conclusion, whether seeds continue or activate dormancy or start germination and seedling establishment under hypoxia depends on the cross-talk of ABA-GA hormones and the regulatory network that leads to the efficient use of seed reserves.

Attempts to decipher the molecular mechanisms that culminate in rice seedlings being able to successfully use starch under anoxia could be incorporated into biotechnological approaches aimed at creating climate-ready crops. In parallel, the identification of barley genotypes characterized by adaptive traits aimed at overcoming limitations of O_2_ is required for genetic improvement programs.

## Figures and Tables

**Table 1:** Rice and barley seed molecular and physiological modification under oxygen deficiency.

Molecular and Physiological Modification	Rice	Barley
**Starch use**	Increased expression and activity of α-amylase under anoxia [15,17]	No α-amylase expression or activity was detected under anoxia [15]
**ABA-GA balance**	Activation of a GA-independent signal under anoxia [26]	Expression of genes involved in GA inactivation and repression of genes involved in GA synthesis under hypoxia [47]
**Pgb/NO cycle**	In deepwater rice, Pgb/NO cycle contributes to ATP generation under anoxia [37]	The over-expression of *Pgb1* supports germination under hypoxia [59]
**N-degron pathway**	-	HvPRT6 is involved in seed germination under hypoxia [82]

## Data Availability

Not applicable.
